# Nobody Loves the Doctor

**Published:** 1914-09

**Authors:** Glenn Robert Guernsey


### Abstracts and Selections.

### Nobody Loves the Doctor.

Nobody loves a doctor,
  Nobody cares for him;
We hate his pills and curse his bills,
  And fear his aspect grim.
He's but a shameful fakir,
  A quack and fraud is he—
But when we're ill, in language shrill,
  We send this C. Q. D.

"Oh, doctor, doctor, doctor!

  Please come right over, quick!

I've a terrible pain in my throbbing brain,
  And I fear I'm mighty sick.
Oh, doctor, doctor, doctor!
  Hurry as fast as you can.
Please hear my cry—I'm about to die—
  And I want a doctor man."

Nobody loves a doctor,
  He cures less than he kills,
For ones he saves, a thousand graves
  With poisoned stiffs he fills.
He is a hardened sinner,
  A liar, a knave and cheat;
Some day he'll die, and then he'll fry,
  But meanwhile we repeat:

"Oh, doctor, doctor, doctor!
  Pray hark to my distress,
My baby's crying, and maybe dying,
  So please don't stop to dress,
Oh, doctor, doctor, doctor!
  I've had an awful fright,
But 'twas that pin that scratched its skin,
  So you can go—Good-night."

Nobody loves a doctor,
  The world would better be
If all his clan, to the very last man,
  Were sunk deep in the sea.
He's but a vain pretender,
  An addle-pated drone,
And yet some day we'll likely say
  These words into a phone:

"Oh, doctor, doctor, doctor!
  Please, doctor, I need you;
The stork is near—it's almost here—
  Oh, doc! what shall I do?
Oh, doctor, doctor, doctor!
  Forgotten are your sins,
If you'll but hurry, I won't worry,
  Not even if it's twins."

—Glenn Robert Guernsey, in *Western Medical Review.*

